# Assessing PRISM context domains and RE-AIM outcomes: Data from use of the Iterative PRISM Webtool

**DOI:** 10.21203/rs.3.rs-7368256/v1

**Published:** 2025-10-08

**Authors:** Russell E Glasgow, Bryan S Ford, Jun Ying, Carlos Rodriguez, Borsika A Rabin, Katy E Trinkley

**Affiliations:** University of Colorado Denver - Anschutz Medical Campus: University of Colorado Anschutz Medical Campus; University of Colorado Denver - Anschutz Medical Campus: University of Colorado Anschutz Medical Campus; University of Colorado Denver - Anschutz Medical Campus: University of Colorado Anschutz Medical Campus; University of Colorado Denver - Anschutz Medical Campus: University of Colorado Anschutz Medical Campus; University of California San Diego; University of Colorado Denver - Anschutz Medical Campus: University of Colorado Anschutz Medical Campus

**Keywords:** PRISM, RE-AIM, implementation outcomes, context, survey, implementation framework

## Abstract

**Background:**

Use of an implementation science (IS) theory, model, or framework (TMF) is one of the hallmarks of a well-executed IS study. While these days TMFs are almost aways used for IS studies, the TMFs themselves are seldom evaluated. Understanding the relationships between the constructs within an IS TMF and their effect on the implementation and effectiveness outcomes can help to refine the TMF, advance IS, and assist implementers.

**Methods:**

We evaluated several hypotheses pertinent to the context domains and Reach, Effectiveness Adoption, Implementation, and Maintenance (RE-AIM) outcomes from the Practical, Robust Implementation and Sustainability Framework (PRISM). Data for these evaluations emerged from the use of the Iterative PRISM (iPRISM) webtool, which includes 21 assessment questions that operationalize PRISM context and outcomes constructs. We tested 11 a priori hypotheses including relationships within and between different framework constructs and considered whether some of the webtool’s assessment questions could be refined or changed to make the assessment more pragmatic.

**Results:**

A total of 348 clinical, community, and public health respondents completed the iPRISM survey as part of using the publicly available webtool. They reported on projects from a wide variety of clinical, community, and public health settings; in English and Spanish; and in different project phases. Seven of the 11 hypotheses were fully or partially supported (e.g., that ratings for Maintenance would be lower than other RE-AIM outcomes). One exception was that the hypothesis that the correlation between Reach and Effectiveness would be the lowest among RE-AIM outcomes was not supported. As hypothesized, scores on the equity items on the various RE-AIM dimensions (e.g., Reach) were consistently lower than general ratings for that dimension. Fewer of the hypotheses about the PRISM context items were supported, possibly due to there being only one item per contextual domain.

**Conclusions:**

The webtool questions provide a standardized way to operationalize PRISM constructs and initial norms for different items. Future research, including qualitative evaluation, is needed to replicate, explore, and understand the complex relationships found within RE-AIM outcomes, within PRISM context domains, and between PRISM context ratings and RE-AIM outcomes.

## Contributions to the literature

- Provides the first large-scale empirical assessment of relationships between contextual domains and RE-AIM outcomes in the Practical Robust Implementation and Sustainability Model (PRISM)
- Evaluates and provides norms on a standard set of pragmatic questions to assess constructs from a leading implementation science framework embedded in a web-based tool
- Tests hypotheses about the relationships among contextual factors; implementation outcomes; and their intersection
- Provides novel data on the incremental value of a set of questions about the equity of implementation outcomes
- Underscores the value of capturing diverse perspectives in implementation planning and evaluation, supporting the use of team-based assessments

## Background

Theories, Models, and Frameworks (TMFs) in implementation science (IS) are a critical means through which we can describe and assess the complex implementation process.^1–3^ When appropriately selected and operationalized, TMFs can ensure that we are addressing the most important implementation questions and considering all pieces of the implementation puzzle including the intervention, implementation strategies, context, and key outcomes.^4^ There is an increasing appreciation for understanding how interventions and implementation strategies need to align with the often dynamically changing implementation context and how they impact the most relevant implementation outcomes when assessing success.^6,7^ Additional questions also remain: how do different elements or dimensions of implementation context interact with each other, what is the relationship among key implementation outcomes, and how do context domains and implementation outcomes relate.^8,9^ Few studies have explored these relationships quantitatively^10–12^ and we have not found any publications assessing these associations longitudinally and across multiple, diverse programs.

Much of the existing literature discusses context domains and implementation outcomes separately. For example, Nilsen and Bernhardsson (2019) conducted a scoping review of 17 determinant frameworks to explore how “context” is operationalized in implementation science and identified common context dimensions but noted considerable inconsistency in terminology and definitions across TMFs.^13^

Other scholars have developed meta-frameworks to integrate contextual domains from multiple TMFs,^14^ explore how one key domain is operationalized across multiple TMFs,^15^ or provide guidance on how to conduct contextual analysis.^16,17^ In parallel, Proctor and colleagues coined definitions for key implementation outcomes^18^ and in a more recent publication discussed nuances associated with their operationalization.^9^ Other work has explored how implementation outcomes across different TMFs can be cross walked to support comparison and synthesis across studies.^19^ There remains a critical gap in our understanding of the dynamic relationships between context and outcomes, and how these connections influence the effectiveness, sustainability, and equity of implementation efforts.

**Figure 1:**
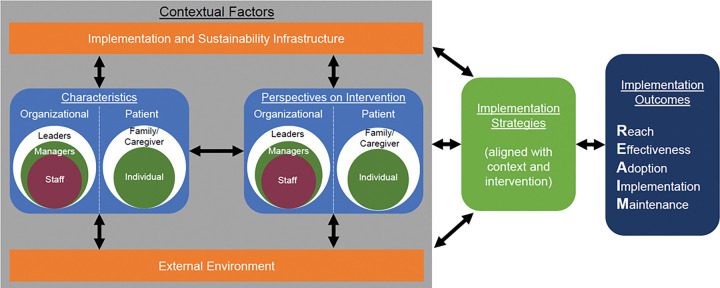
The Practical, Robust Implementation and Sustainability Model (PRISM)

To explore the above outlined questions about complex relationships, it makes sense to look at data from studies that used one of these TMFs (or a combination of TMFs that involve both context and outcomes). One such example is the Practical, Robust Implementation and Sustainability Model (PRISM) which includes both multilevel and multi-perspective consideration of the implementation context through the PRISM context domains as well as a set of implementation (RE-AIM) outcomes.^8,22,23^ PRISM was developed to address specific contextual factors related to successful adoption, implementation, and sustainability. PRISM includes RE-AIM (Reach, Effectiveness, Adoption, Implementation, and Maintenance) outcomes and is a pragmatic, contextual expansion of the widely used RE-AIM framewo*rk*.^8,22–24^ It focuses on four contextual factors that influence RE-AIM implementation outcomes. As shown in Fig. 1, these factors are: organizational and patient perspectives on the intervention; organizational and patient characteristics; external environment factors (e.g., reimbursement policies, reporting guidelines), and the implementation and sustainability infrastructure (e.g., resources, aligned organization priorities, audit and feedback processes). While PRISM was created on the premise that context predicts or is associated with implementation and effectiveness outcomes, this presumed relationship has not been formally tested to our knowledge.

The purposes of this paper are to: (1) describe and summarize data collected on PRISM contextual domains and RE-AIM outcome variables; (2) investigate specific hypotheses concerning relationships among and between contextual and outcome constructs; (3) examine implications for refining a standard set of assessment items for the Iterative PRISM (iPRISM) Webtool^26^ to enhance their pragmatic utility; and (4) recommend directions for future research on PRISM and related frameworks. Specific hypotheses and research questions were defined a priori and are detailed in the Methods section and Table 3. The overarching aim was to deepen understanding of how PRISM domains interact. This will inform both the refinement of the iPRISM Webtool and the development of a standardized, pragmatic set of items to operationalize PRISM constructs and outcomes.

## Methods

Data were collected using the iPRISM Webtool,^24,26^ which is freely and publicly available for researchers and implementers to use. The iPRISM Webtool was developed to increase accessibility of PRISM, thus is available in English and Spanish and is designed to be used by a broad audience, including those without IS expertise. The electronic format facilitates real time prompts and guides users through the process of applying PRISM to their projects and includes embedded education. It can be used by individuals or teams, with each member providing their independent perspective on the PRISM factors. When used as a team, individual responses are collated and summary feedback provided.

The iPRISM Webtool operationalizes PRISM using 21 assessment questions that are mapped on the PRISM contextual domains and RE-AIM outcomes (see Additional File 1). It is designed to be used across all phases of a project. Each assessment question is rated on a 6-point sliding Likert scale anchored by the terms from 1= ‘not at all likely’ to 6= ‘very likely’ for RE-AIM items and from 1= ‘not aligned’ to 6= ‘very aligned’ to the question ‘How aligned is your program with (PRISM characteristic)?’ and has an option to select not applicable. Except for Implementation, all of RE-AIM dimension assessment questions have both a general question and a question to assess representativeness or equity on that dimension. Users can decide whether to stop after completion of the assessment questions and feedback; or continue with a section that guides users through the process of identifying and prioritizing implementation strategies (adaptations) to optimize the contextual alignment and/or enhance the impact of their project on RE-AIM outcomes. All responses and data entered in iPRISM are anonymous, but general characteristics about the project and person completing it are optional fields.

This study was classified as exempt by the Colorado Multiple Institutional Review Board under protocol number 23–0179.

### Hypotheses and Rationales

Because of the large number of potential analyses, comparisons and correlations, we made a priori hypotheses about 11 specific issues. These hypotheses were based on the purpose of PRISM and the authors’ experience developing and using PRISM and its RE-AIM dimensions spanning 25 years of public health and IS research. After two rounds of discussion, final hypotheses were selected after reaching consensus among the authors. We also developed three other key Research Questions that did not have specific hypotheses.

### Scores on RE-AIM dimensions and PRISM domains

We developed four hypotheses and one general research question involving comparisons of average scores on RE-AIM dimensions and alignment scores on PRISM domains.

#### Ho 1:

The mean value on the Maintenance dimension will be lower than the other RE-AIM dimensions (i.e., Reach, Effectiveness, Adoption, Implementation). Rationale: Maintenance is contingent on success at earlier stages and notoriously difficult to achieve.

#### Ho 2:

The mean score on Implementation and Sustainability Infrastructure (ISI) will be lower than other PRISM context domains. Rationale: Resources are frequently limited, especially in low-resource settings and few organizations have robust procedures for audit and feedback.

#### Ho 3:

Among RE-AIM dimensions, mean scores for the Reach and Adoption dimensions will correlate most highly with each other; and Reach and Effectiveness the lowest. Rationale: Definitions of Reach and Adoption are the same, just at different levels. Often actions taken to maximize effectiveness can limit reach, and vice versa.

#### Ho 4:

Relationships among the three components of Implementation: fidelity, adaptation, and costs will be only modestly correlated (e.g., 0.3 or lower). Rationale: These are different constructs and are not expected to be strongly related, but conceptually all are part of the multifaceted construct of implementation.

Exploratory Research Question 1: What are the correlations among PRISM context measures? We believe this is an important issue to explore that could advance our understanding and assessments.

### Relationships between General RE-AIM and RE-AIM Equity Questions

#### Ho 5:

Scores on the representativeness/equity items for each RE-AIM dimension (e.g. Reach) will be lower than the general item for that dimension. Rationale: It is more challenging to produce results that are similar across all groups given societal, structural and social needs issues,^27–29^ than to produce a good overall (average) outcome.

#### Ho 6:

There will be a high correlation between equity and general scores- (r = .6 or higher) on each RE-AIM dimension. Rationale: As the questions relate to the same construct (e.g., Reach) they should be strongly related, although the mean level on the equity items are hypothesized to be lower (Ho 5).

#### Ho7 :

Responses to the RE-AIM equity items will be significantly related to each other. Rationale: a) Programs that produce differential results across one dimension will be likely to also do so on other issues and b) respondents are likely to conceptualize equity as a global issue.

### Relationships between PRISM context domains and RE-AIM outcomes

#### Ho 8:

Organizational and recipient perspectives on the intervention/implementation strategy will be most strongly related to Adoption and Maintenance among the RE-AIM dimensions. Rationale: Experiences of a) organizations and b) individual recipients with similar programs in the past are likely to heavily influence adoption decisions and propensity to continue a program after research support is no longer available.

#### Ho 9:

Patient (recipient/participant) Characteristics will be more strongly related to Reach than other RE-AIM dimensions. Rationale: Individual characteristics include social and structural factors that can heavily influence willingness to participate in a program.

#### Ho 10:

Implementation and Sustainability Infrastructure (ISI) will be more strongly related to Implementation and Maintenance than other RE-AIM outcome dimensions. Rationale: As its title implies, the ISI domain was developed to include factors presumed to strongly impact these RE-AIM outcomes.

#### Ho 11:

External Environment scores will most highly correlate with Adoption and Maintenance among the RE-AIM outcome dimensions. Rationale: Higher level policy, structural and incentive issues included in the External Environment domain of PRISM should strongly influence organizational Adoption and Maintenance decisions.

### Congruence among team members on same project

Exploratory Research Question 2: Do implementers and front-line staff have lower ratings (less positive) than Investigators or Supervisors? Rationale: This question has implications for future implementation, but we do not have specific hypotheses.

Exploratory Research Question 3: How much congruence is there among team members? Rationale: This has implications for future implementation research and practice, including use of the team function on the iPRISM webtool.

### Analyses

Outcomes of likelihood scores on RE-AIM dimensions and alignment scores on PRISM domains were summarized using means (standard deviation or standard error) and medians. To compare likelihood scores among RE-AIM dimensions, a fixed effect model was used. For specific hypotheses, mean differences were evaluated using p < .01 level of significance. For comparisons of RE-AIM dimension general scores versus the equity scores, a mixed effect model was applied, using the likelihood score as the dependent variable, RE-AIM dimension, equity (general vs. equity), and their interaction as the fixed effects, and a random effect of participants to account for the within person correlation.

To assess the associations of likelihood score of RE-AIM or alignment score of PRISM to characteristics of institutional settings, roles of participants, and stages of implementation, fixed effects models were applied using the setting (or role, or stage), the dimension (or domain), and their interaction as the fixed effects of interest. Means were compared between settings (or roles, or stages) under each dimension (or domain). As sensitivity analysis, non-parametric Wilcoxon rank sum tests and Wilcoxon signed rank tests were compared to results from fixed effect models and mixed effect models respectively. Those results were not reported unless they did not agree with the parametric findings. For relationships among RE-AIM dimensions, PRISM domains, and RE-AIM dimensions vs. PRISM domains, Pearson’s correlation coefficients were used. Spearman’s correlation coefficients were used as sensitivity analysis. For within team correlations, a random effect model was used for each RE-AIM dimension score (or PRISM domain) score, the intraclass correlation coefficient (ICC) was estimated from the proportion of the variance of the random effect to the total variance. A 95% confidence interval of the ICC was computed.

Missing observations were evaluated for mechanism before formal statistical analysis and considered as missing completely at random (MCAR) or missing at random (MAR). Maximum likelihood methods were used in all parametric statistical models in analyses to handle the missing data problems. All statistical analyses were performed using SAS software (SAS, Cary, NC) and R software. Correlations were considered as “Very Strong”, “Strong”, “Moderate”, “Mild”, “Weak” and “Very Weak” if correlation coefficients (r) were > 0.75, 0.65–0.74, 0.55–0.64, 0.45–0.54, 0.35–0.44, and < 0.35 respectively.

## Results

### Survey Respondent and Project Characteristics

Between January 10, 2023, and January 7, 2025, 348 users completed the tool. Of those users, 182 (52.3%) answered as members of a team; 155 (44.5%) reported as the sole representative of their program; and 11 (3.2%) did not select team or individual. There was representation from all professional roles with Researcher being the most often selected, 133 (38.2%), and Clinician the next most common, 108 (31.0%). A wide variety of settings were represented with 201 (57.8%) coming from Clinical settings, 141 (40.5%) from Community settings, and 124 (35.6%) from Public Health settings. The questions were completed in English by 310 (89.1%) users and in Spanish by 38 (10.9%) users. The Stage of the projects were reported as 155 (44.5%) in Planning, 131 (37.6%) in Implementing, 57 (16.4%) in Sustaining. Table 1 provides more details.

### Descriptive Data

Table 2 presents descriptive data on the iPRISM assessment questions, including number of respondents, the mean, standard deviation, range and percentage of respondents selecting the maximum value for each item. Most items received moderate to high scores (average of slightly over 4 on the 6-point scale), with small to moderate standard deviations.

### Results of Hypothesis Testing

#### Ho 1:

The mean value on Maintenance will be lower than other RE-AIM dimensions. This hypothesis was partially confirmed with a caveat (Table 3). The average Maintenance score was lower than three of the 4 general RE-AIM scores. The average Reach score was slightly lower than the Maintenance score: 4.22 vs. 4.26. The only significant comparison at p < .01 involving Maintenance was that the Maintenance score was lower than the Implementation adaptation component of Implementation (p<. 007), although Maintenance scores were marginally lower than Effectiveness scores (p < .05).

#### Ho 2:

The mean score on Implementation and Sustainability Infrastructure (ISI) will be lower than other PRISM context domains.

This hypothesis was supported. The ISI score (mean 3.96) was the lowest individual PRISM domain score, and significantly lower (p < .0001) than all other PRISM context domains except for External Environment (mean 4.06).

#### Ho 3:

**Figure 2:**
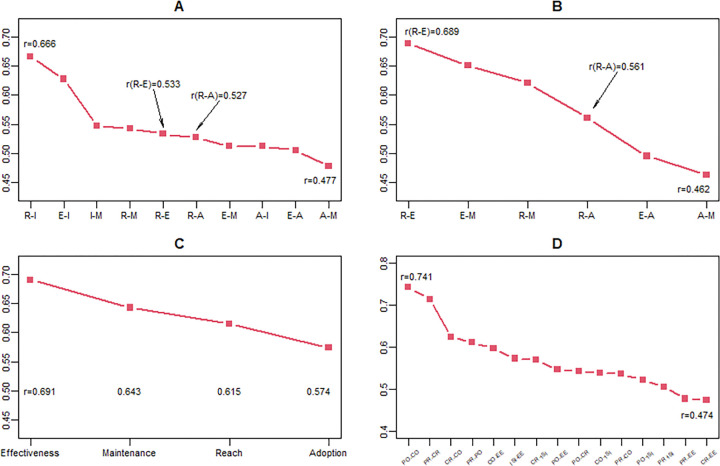
Correlations among RE-AIM dimensions and PRISM domains (A) Correlation coefficients among general RE-AIM dimension scores (B) Correlation coefficients among RE-AIM equity scores (C) Correlation coefficients between RE-AIM general and equity scores (D) Correlation coefficients among PRISM domain scores In RE-AIM dimensions, R = Reach, E = Effectiveness, A = Adoption, I = Implementation, and M = Maintenance. In PRISM domains, CO = Characteristics of Organization, CR = Characteristics of Recipients, EE = External Environment, EO = Perspectives of Organization, ER = Perspectives of Recipients, and ISI = Implementation Sustainability Infrastructure.

Among RE-AIM dimensions, scores for the Reach and Adoption will correlate most highly with each other and Reach and Effectiveness the lowest. This multi-part hypothesis was rejected, and some results were in the direction opposite to that predicted (see Table 3). Implementation and Reach had the highest correlation (r=. 67), followed by Implementation and Effectiveness (r = .63). Adoption and Maintenance had the lowest correlation (r = 0.48). All RE-AIM scores were moderately correlated (range r = .48- .67). Contrary to prediction, the Reach-Effectiveness correlation was not the lowest, but intermediate in magnitude among the intercorrelations of RE-AIM outcome dimensions (r = .53; see Fig. 2).

1. Correlation coefficients among general RE-AIM dimension scores
2. Correlation coefficients among RE-AIM equity scores
3. Correlation coefficients between RE-AIM general and equity scores
4. Correlation coefficients among PRISM domain scores

In RE-AIM dimensions, R = Reach, E = Effectiveness, A = Adoption, I = Implementation, and M = Maintenance.

In PRISM domains, CO = Characteristics of Organization, CR = Characteristics of Recipients, EE = External Environment, EO = Perspectives of Organization, ER = Perspectives of Recipients, and ISI = Implementation Sustainability Infrastructure.

#### Ho 4:

Relationships among the three components of Implementation: fidelity, adaptation, and costs will be only modestly correlated (e.g., 0.3 or lower). This hypothesis was not supported. All subcomponents of Implementation were moderately and significantly intercorrelated (r = .46- .53, p < .01).

### Research Question 1: What are the correlations among PRISM context measures?

All six PRISM domain scores were moderately to highly correlated, r = .48-.74 (Fig. 2), and all correlations were highly significant (all p < .0001). The two highest correlations were between the two Organization domains (Perspectives and Characteristics) r = .74 and between the two ‘Recipient’ (e.g., patients) domains, r = .71.

### Relationships between General RE-AIM and Equity RE-AIM Questions

#### Ho 5:

Scores on the equity items for each RE-AIM dimension (e.g. Reach) will be lower than the general items. This hypothesis was supported. As shown in Tables 2 and 3, on RE-AIM dimensions, the equity item had lower average scores than the general item (there was not an equity item for Implementation). These differences were generally moderate given the standard deviations and ranged from 0.2 to 0.51 points. All except the comparison between general Reach and Reach equity scores (4.22 vs. 4.06, p < .04) were statistically significant.

#### Ho 6:

There will be a high correlation between the equity items and the general item (r = .6 or higher) on each RE-AIM dimension. This hypothesis was partially supported (Table 3). The equity and general RE-AIM scores on the various dimensions were moderately to strongly correlated, r = .57- .69 (all p < .001) although only 3 of 4 exceeded .60 (see Fig. 2).

#### Ho7:

Responses to the four ‘equity’ items will be significantly related to each other. This hypothesis was supported. All equity scores were significantly correlated (r = .46-.69, p < 0001: see Fig. 2).

### Relationships between PRISM domains and RE-AIM outcomes

#### Ho 8:

**Figure 3:**
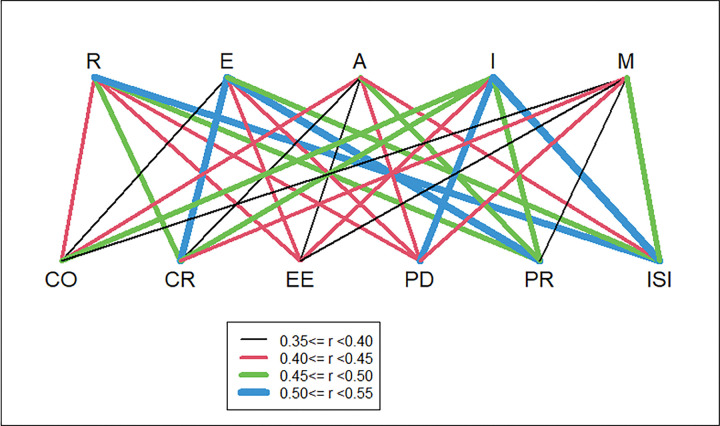
Correlation coefficients between RE-AIM dimensions and PRISM domains In RE-AIM dimensions, R = Reach, E = Effectiveness, A = Adoption, I = Implementation, and M = Maintenance. In PRISM domains, CO = Characteristics of Organization, CR = Characteristics of Recipients, EE = External Environment, EO = Perspectives of Organization, ER = Perspectives of Recipients, and ISI = Implementation Sustainability Infrastructure.

PRISM organizational and recipient perspectives will be more strongly correlated with Adoption and Maintenance than with other RE-AIM dimensions. This hypothesis was not confirmed (Table 3). All correlations were significant but the correlation between PRISM Organizational and Recipient Perspective domains and Adoption and Maintenance were among the lowest correlations (r = 0.28 and 0.46; Fig. 3).

In RE-AIM dimensions, R = Reach, E = Effectiveness, A = Adoption, I = Implementation, and M = Maintenance.

In PRISM domains, CO = Characteristics of Organization, CR = Characteristics of Recipients, EE = External Environment, EO = Perspectives of Organization, ER = Perspectives of Recipients, and ISI = Implementation Sustainability Infrastructure.

#### Ho 9:

Recipient (patient/participant) Characteristics will be more strongly related to Reach than other RE-AIM dimensions. This hypothesis was partially supported (Table 3 and Fig. 3). The correlation with Reach (r = 0.48, p < .001) was the second highest association with Recipient Characteristics. Recipient Characteristics was most correlated with Effectiveness (r = 0.51) and least correlated with Adoption (r = 0.40).

#### Ho 10:

ISI will be more highly correlated with Implementation and Maintenance than other RE-AIM dimensions. ISI was most strongly related to Implementation (r = 0.54), followed by Reach (0.52) and then maintenance (r = 0.50). Thus, this hypothesis was partially supported with the caveat that correlations between all ISI and all RE-AIM outcomes were moderate.

#### Ho 11:

External Environment scores will be most highly correlated with Adoption and Maintenance. This hypothesis was not supported (Table 3). The correlation between External Environment and Adoption (r = .35) was lower than with other RE-AIM dimensions. External Environment correlated r = .40 with Maintenance, but this was less than with 3 other RE-AIM outcomes.

### Questions about Responses from Team Members Completing iPRISM

Exploratory Research Question 2: Do implementers and front-line staff provide lower ratings (less positive) than investigators or supervisors? This question needed to be modified given that respondents were able to select multiple options for their role and many did so. Due to insufficient numbers of respondents who were clearly ‘implementers’ vs. investigators, it was not possible to compare these groups directly. The related question we were able to answer was “Were ratings from a) clinicians (n = 108) and from b) researchers (n = 133) higher or lower than those from those in all other roles combined?”

Clinicians saw programs more positively (rated higher) than those in other roles on all 18 items, often by approximately a half point. On 10 of 18 comparisons (including both PRISM context and RE-AIM outcomes) clinician ratings were significantly higher at the p < .01 level and 5 other comparisons were marginally significant. In contrast, ratings from researchers were similar to or slightly lower than those in other roles. Only 1 of 18 comparisons was significant, and the magnitude of differences was small.

Exploratory Research Question 3: How much congruence was there among team members? The sample size for this analysis was restricted to 22 teams consisting of a total of 127 raters. Intraclass correlations (ICCs) revealed low levels of agreement on how different team members rated the items. ICCs for PRISM context variables were 0.14–0.20; none were significant at p < .01. ICCs for RE-AIM outcomes items were slightly higher with a median of .23, and 5 of 12 were significant at p < .01.

Additional Research Question: How did ratings vary across project phases? One additional question emerged during our analyses. Although most respondents completed the webtool during the planning stages (n = 157), there were enough who completed the survey during implementation (n = 122) or sustainment phases (n = 56) to conduct exploratory analyses. Both RE-AIM outcomes and program alignment with PRISM contextual factors were consistently lower during the implementation phase than during the planning or sustainment phases. On all 18 items, ratings were lower during implementation than during planning or sustainment phases. With one exception, every comparison between the implementation phase and each of the other phases was significant at p<. 05 or less, usually at the p < .0001 level. Mean ratings between the planning and sustainment phases did not differ.

## Discussion

To our knowledge, this was the largest study to date evaluating assessment items related to PRISM and the first to assess the relationship between contextual and outcome constructs of an IS TMF. These data from the sizable and moderately diverse sample of implementation and health services researchers and practitioners in this report provides initial norms for the PRISM assessment items. These questions (Appendix) can be used via the iPRISM Webtool or separately. This evaluation serves to not only refine a standardized set of assessment questions that operationalize one TMF; the findings can also be used to inform other IS TMFs and implementation science approaches that aim to pragmatically improve contextual fit and optimize equitable outcomes of evidence-based programs. While TMFs are used in the majority of IS papers and grants, they are seldom if ever evaluated to understand the complex interactions between the different elements of implementation context and outcomes.^1,10,13,30^ This evaluation represents one such effort.

The scope of this study was limited to PRISM-defined categories of context and its RE-AIM implementation outcomes. These factors may be labeled differently across other IS TMFs, but many constructs are similar and can be cross walked.^2,19^ Although PRISM is a framework, not a formal theory, there is sufficient information about its purpose, development and use to justify forming hypotheses.. Further research is needed to understand the complex relationships between the dynamically changing context and outcomes in real world settings.

There were some key findings related to the RE-AIM equity specific questions. As predicted, ratings on the RE-AIM equity items were consistently lower than the general RE-AIM ratings and there were moderate to high correlations between the equity components and the general dimensions. Given these results, we considered whether the equity items on the iPRISM Webtool add sufficient value that they should be retained. Although the differences were small, the equity items did significantly differ from their respective general RE-AIM dimension score, and the correlations of .55 to .66 only account for 35–43% of the variance in equity ratings. Our decision, pending further data, is to allow programs to decide whether. to include the equity questions, but to recommend they do.

This study also explored the relationship between PRISM contextual domains and implementation outcomes.^8,22^ Two of four hypotheses about contextual factors were moderately supported (including multi-part predictions summarized in Table 3). As predicted, ISI ratings were strongly related to the RE-AM Implementation and Maintenance outcome dimensions. We also hypothesized PRISM’s Recipient Characteristics would be most strongly correlated with Reach but found that it was most correlated with Effectiveness (r = 0.51), followed by Reach (r = 0.48). The two hypotheses that were not supported were that 1) the two PRISM ‘perspective scores’ (Organizational and Recipient) would be more highly related to Adoption and Maintenance than other RE-AIM outcomes; and 2) External Environment ratings would be most highly related to Adoption and Maintenance.

Overall, the correlations between ratings of contextual alignment and RE-AIM outcomes were moderate. Conceptually, these correlations would not be predicted to be much higher due to other key factors (moderators and mediators) of different problems being addressed, implementation strategies used, types of interventions, and different settings and populations. In addition, having only 1–2 items per construct substantially diminishes the chances of finding strong relationships with a given construct.

It is notable that responses a) within the RE-AIM outcome dimensions and b) within the PRISM context domains were moderately correlated, the PRISM context ratings more so than the RE-AIM ones. Although this was not a specific prediction, it makes sense given that neither RE-AIM outcome nor PRISM context constructs were ever conceptualized to be independent.^22,24,31^ The PRISM context ratings were all significantly correlated, with the strongest correlations between the Perspectives and Characteristics within both levels of the Recipients (r = 0.71) and the Organization (r = 0.74). We considered whether to keep the Perspective and Characteristic items as separate assessment questions, and whether to reword them, given these high correlations. We recommend keeping them as separate ratings for now, and to qualitatively assess respondents’ thought processes in answering these questions to make a more informed decision. In other work evaluating a set of 29 items assessing the PRISM context questions, there was moderate internal consistency within the 3–6 items comprising each of the PRISM domains.^33^

Our hypotheses concerning relationships among the RE-AIM outcome dimensions were partially supported. We hypothesized that the correlation between Reach and Effectiveness would be the lowest based on our experience with ‘objective data’ in prior research,^34^ but this was not what we found. It is possible that in the absence of more detailed information on the definition of different PRISM and RE-AIM terms that respondents have a general impression about the program they are implementing and do not differentiate sharply among the different PRISM factors. The components of implementation – cost, adaptation and fidelity were significantly correlated, perhaps because they may be perceived as related, given they are part of the same overarching outcome dimension. In future iterations of the PRISM assessment items, we will consider collapsing the 3-part implementation items into one to improve pragmatism. As predicted, mean ratings for Maintenance were lower than the composite of the other four RE-AIM dimensions; this may be in part due to most respondents being in the planning stage of their project or that sustainability of the project was not prioritized.

Although we did not articulate a specific research question about ratings across project phases a priori, these analyses produced very clear and consistent findings. Mean ratings across all constructs were lower for the Implementation (program delivery) phase than either Planning or Sustainment. This is likely because of the high probability of encountering numerous barriers to program delivery during the Implementation phase.^35^ In the future, with larger sample sizes we will evaluate the relationship between the PRISM context factors and RE-AIM outcomes within each project phase.

We found that individual team members responded differently to the assessment items. This lack of agreement across team members does not necessarily imply low reliability but is likely a reflection of different individuals observing different issues and outcomes. These differences among individuals within teams support the importance of partner engagement across levels and seeking diverse representation.^27–29^ These findings also support the team-based option of the iPRISM Webtool.

### Limitations and strengths

This was an initial study of data derived from the use of the iPRISM Webtool.^26^ When interpreting these results, it is important to keep in mind that the brief iPRISM assessment items are not intended to be a psychometrically elegant survey, but to serve a very practical purpose. The assessment items and the iPRISM Webtool were designed to be very brief to maximize ease of use and obtain high completion rates. Their purpose is to assess progress, identify areas for improvement and to facilitate discussion among team members to identify implementation strategies.^26,36^ Further, because responses consist of rater perceptions, there are no objective data in this study against which to validate the ratings. Thus, we did not conduct standard psychometric analyses, especially as iPRISM had only one (for context measures) or two items (for implementation outcomes) per construct.

There are several limitations, as well as strengths, to our analyses and paper. Limitations include not having information on respondents other than their role in the project. The nature of the sample that consisted of users from several different countries, from vastly different projects made it difficult to compare ratings. There were also many analyses conducted and despite our attempting to mitigate this by adopting a moderately conservative alpha and specifying a priori hypotheses, some findings may be due to chance. Comparisons of results across phases were cross sectional, and respondents provided data for only one time point, rather than at multiple time points.

While many relationships were statistically significant, due to the relatively large sample size, the magnitude of effect was often not large. We also observed a modest ceiling effect (from 7–26% used the maximum rating of 6, median = 18%) among survey items. There were relatively few missing data, but slightly more missing responses on the PRISM context and RE-AIM equity questions than the standard RE-AIM items (approximately 9 versus 5%), suggesting that these items may have been less clear or more difficult to answer. Our findings are also dependent on the respondents’ interpretation of the assessment questions and the standard PRISM definitions may have been understood differently across projects. For example, the originally defined RE-AIM dimension of adoption has been revised for specific projects.^37,38^

Strengths of the study include the large sample size and the small amount of missing data especially in the context that there were no incentives or reminders for survey completion, and that this was a naturalistic assessment, not a formal research study. To our knowledge this is the first project to provide standard survey items to obtain respondent perceptions of both PRISM context and its RE-AIM outcome variables, or to empirically study their relationship.

## Conclusions

We conclude that this relatively brief set of questions assessing many components of PRISM is an appropriate, relatively brief, pragmatic set of items for the purposes of the Iterative PRISM webtool. Prospective use of the iPRISM Webtool in a large project with a standard intervention and objective data on at least some RE-AIM outcomes (e.g., participation rate, implementation fidelity, heterogeneity of outcomes), would facilitate understanding of the usefulness of these subjective ratings. Research is needed on the use of the webtool over time; The incremental value of the equity-specific RE-AIM items and the three component Implementation items deserve further study.

Finally, there is a need for continual evaluation of IS TMFs, including PRISM, to understand the complex relationships between implementation contexts and outcomes. Such evaluations are important to enhance pragmatism of IS efforts to align with context.^39^ They can also help to study the importance of assessing all constructs in a TMF and if some constructs that may be more essential than others. Enhancing the pragmatism of IS TMFs, including brief standardized assessment items that operationalize TMFs can assist in making IS methods more accessible for broad use.

In conclusion, we encourage replication of our findings and further research to evaluate IS TMFs and pragmatic assessment tools to operationalize TMFs for both practice and research. Further investigation of the relationship of PRISM and other TMF contextual factors to implementation and clinical effectiveness outcomes can help advance the field.

## Supplementary Material

Supplementary Files

This is a list of supplementary files associated with this preprint. Click to download.

• AddtlFile1iPRISMWebtoolsurveyitems7.15.25.docx

• AddtlFile2iPRISMSTROBEchecklistcrosssectionalfinal.docx

## Figures and Tables

**Table 1.** Characteristics of iPRISM users

Characteristic	N of 348 (%)
**-**	**-**
**Team/Individual**	-
Team	182, (52.3%)
Individual	155, (44.5%)
Missing	11, (3.2%)

**Professional Role** (could select more than 1)	
Researcher	133, (38.2%)
Clinician	108, (31.0%)
Other	46, (13.2%)
Program manager	39, (11.2%)
Public health practitioner	37, (10.6%)
Implementation specialist	30, (8.6%)
Quality improvement specialist	21, (6.0%)
Clinical administrator (e.g., medical director)	15, (4.3%)
Academic administrator (e.g., department chair, dean)	11, (3.2%)

**Setting** (could select more than 1)	
Clinical	201, (57.8%)
Community	141, (40.5%)
Public health	124, (35.6%)
Other	27, (7.8%)

**Language**	
English	310, (89.1%)
Spanish	38, (10.9%)

**Stage**	
Planning	155, (44.5%)
Implementing	131, (37.6%)
Sustaining	57, (16.4%)
Missing	5, (1.5%)

**Table 2.** Summary statistics for PRISM and RE-AIM measures (N=348)

Measure	N	Mean (STD)	Median	% response with highest score
***PRISM context measures***				
Perspectives of Recipients	317	4.3 (1.3)	4.0	20.8
Perspectives of Organization	317	4.3 (1.2)	4.0	18.6
Characteristics of Recipients	316	4.3 (1.3)	4.0	19.3
Characteristics of Organization	316	4.3 (1.3)	4.0	21.5
Implementation Sustainability Infrastructure	318	4.0 (1.3)	4.0	13.8
External Environment	316	4.0 (1.3)	4.0	16.5
***RE-AIM outcome measures***				
Reach	330	4.2 (1.3)	4.0	19.7
Effectiveness	331	4.4 (1.3)	5.0	21.8
Adoption	331	4.3 (1.4)	4.0	21.5
Implementation Composite	332	4.3 (1.1)	4.3	11.75
Implementation Adaptation	334	4.5 (1.3)	5.0	26.1
Implementation Cost	325	4.3 (1.3)	4.0	19.7
Implementation Fidelity	329	4.2 (1.3)	4.0	16.1
Maintenance	328	4.3 (1.2)	4.0	18.3
***RE-AIM equity measures***				
Reach Equity	315	4.1 (1.4)	4.0	17.8
Effectiveness Equity	312	4.2 (1.3)	4.0	18.3
Adoption Equity	316	3.9 (1.4)	4.0	15.8
Maintenance Equity	311	4.1 (1.3)	4.0	17.4

Footnote: The response range for all items was 1–6. ‘Implementation Composite’ is the mean of all implementation items creating a continuous scale for this one item only.

**Table 3.** Results Related to Hypotheses and Key Questions Based on Iterative PRISM Webtool Data

Overall Scores and Mean Differences within RE-AIM and PRISM constructs		
Hypothesis	Result	Interpretation
***RE-AIM dimensions and PRISM domains***		
1.Maintenance mean score will be lower than the other RE-AIM dimensions *Partially supported*	Mean (SD) Likert scores: **M**aintenance, 4.26 (1.24) Mean composite score of other 3 RE-AIM dimensions, 4.3 **R**each, 4.22 (1.34) **E**ffectiveness, 4.4 (1.29) **A**doption, 4.26 (1.35) **I**mplementation, 4.3 (1.06)	Reach was the lowest followed closely by maintenance and adoption The magnitude of difference in mean scores was small
2. Implementation Sustainability Infrastructure (ISI) mean score will be lower than other PRISM domains *Supported*	Mean (SD) Likert scores: ISI, 3.96 (1.27) Recipient perspectives, 4.28 (1.28) Organization perspectives, 4.3 (1.23) Recipient characteristics, 4.28 (1.25) Organization characteristics, 4.31 (1.27) External Environment, 4.06 (1.34)	ISI was lower than other domains The magnitude of difference in mean scores was small to moderate
3a. Reach and Adoption will have the highest correlation among the RE-AIM dimensions *Not Supported*	Correlation between select RE-AIM dimensions: **I**mplementation and **R**each, r=0.67 p < 0.0001) **I**mplementation and **E**ffectiveness, r=0.63 (p <0.0001) **I**mplementation and **M**aintenance, r=0.55 (p <0.0001) **R**each and **E**ffectiveness, r=0.53 (p <0.0001) **R**each and **A**doption, r=0.53 (p <0.0001) **A**doption and **M**aintenance, r=0.48 (p <0.0001) Range across all dimensions, r= 0.48–0.67	Implementation and Reach were most highly correlated The second highest correlation was Implementation and Effectiveness Adoption and Maintenance were least correlated Strength of correlations were moderate
3b. Reach and Effectiveness will have the lowest correlation among the RE-AIM dimensions *Not Supported*		
4. Relationships among the 3 Implementation components will be non-significant (r≤ 0.3) *Not supported*	Correlations were: Fidelity- Cost, r= .47 Fidelity- Adaptation, r= .53 Cost- Adaptation, r= .46	Correlations were moderate and somewhat higher than expected. They were significant although likely due, in part, to large sample size. *Not supported*
Research Question 1-What are the correlations among the PRISM domains?	Correlation between select PRISM domains: Organizational perspectives and characteristics, r=0.74 Recipient perspectives and characteristics, r=0.71 Recipient perspectives and external environment, r=0.48; was the lowest correlation Range across all domains, r=0.48–0.74 (all p<0.0001)	All relationships were statistically significant; Strength of correlations were moderate to high, and correlations between perspectives and characteristics within a ‘level’ (e.g. Organization) were substantially higher than other correlations, but all significant.
***Relationships between general RE-AIM dimensions and equity sub-items***		
5. Mean equity scores will be lower than for the general score for each RE-AIM dimension *Supported*	Equity scores per dimension / General score per dimension, mean (SD): **R**each, 4.06 (1.34)/4.22 (1.37) **E**ffectiveness, 4.21 (1.29)/4.4 (1.33) **A**doption, 3.9 (1.35)/4.26 (1.42) **I**mplementation, n/a **M**aintenance, 4.09 (1.24)/4.26 (1.33)	Equity scores were all lower than the general dimension scores. Mean differences were modest but statistically significant (p<.001) except for the Reach dimension (p<.04). The largest difference was with adoption
6. Will be a high correlation between the equity score and the general score for each RE-AIM dimension (.6 or higher) *Supported*	Correlation between equity and general scores for each RE-AIM dimension: **R**each, r=0.62 **E**ffectiveness, r=0.69 **A**doption, r=0.57 **I**mplementation, n/a **M**aintenance, 0.644 (all p=<0.0001)	All relationships were statistically significant: the strength of correlations were moderate to strong and similar in magnitude
7. Different ‘Equity scores’ will be significantly related to each other *Supported*	All correlations were between r = .46 (Adoption- Maintenance) and .69 (Reach- Effectiveness) All p < .001	Correlations were moderate to high, and all highly significant (p<.001)
***Relationships between PRISM and RE-AIM***		
8. PRISM’s Organizational and Recipient Perspective domains will be more correlated with RE-AIM’s Adoption and Maintenance dimensions than other RE-AIM dimensions *Not supported*	Correlations between PRISM’s Organization and Recipient Perspectives domains and select RE-AIM dimensions: Recipient perspective and **E**ffectiveness, r=0.521 Organization perspective and **I**mplementation, r=0.506 Recipient perspective and **A**doption, r=0.458 Organization perspective and **A**doption, r=0.423 Recipient perspective and **M**aintenance, r=0.389 Organization perspective and **M**aintenance, r=0.284	Lower correlations with Maintenance than other RE-AIM dimensions Recipient perspective was most correlated with Effectiveness Organization perspective was most highly correlated with Implementation
9. PRISM’s Recipient Characteristic domain will be more correlated to Reach than other RE-AIM dimensions *Partially supported* (2^nd^ highest)	Correlation between PRISM’s Recipient Characteristic domain and RE-AIM dimensions: Recipient characteristics and **E**ffectiveness, r=.51 Recipient characteristics and **R**each r=.48 Recipient characteristics and Implementation, r= .46 Recipient characteristics and **M**aintenance, r=.41 Recipient characteristics and **A**doption, r=0.40	Recipient characteristics was most correlated with effectiveness, followed by reach, but the absolute magnitude of difference in correlation was small
10. PRISM’s ISI domain will be more correlated with RE-AIM’s Implementation and Maintenance dimensions than other RE-AM dimensions *Partially supported*	Correlation between PRISM’s ISI domain and select RE-AIM constructs: ISI and **I**mplementation, r=.54 ISI and **R**each, r=.52 ISI and **M**aintenance, r=.50 ISI and **E**ffectiveness, r= .46 ISI and **A**doption, r= .43	ISI was most correlated with Implementation followed by Reach and then Maintenance Absolute magnitude of difference was small
11. PRISM’s External Environment domain will be more highly correlated with RE-AIM’s Adoption and Maintenance dimensions than other REAM dimensions *Not supported*	Correlation between PRISM’s External Environment domain and select RE-AIM constructs: External Environment and **I**mplementation, r=0.43 External Environment and **R**each, r= .42 External Environment and **E**ffectiveness, r=.41 External Environment and **M**aintenance, r=.40 External Environment and **A**doption, r=.35	External Environment was most correlated with Implementation and least correlated with Adoption Absolute magnitude of difference was small
***Congruence Among Team Members and Across Project Phases***		
Research Question 2: Are ratings from ‘Implementers’ lower than those from Investigators	Clinicians (n = 108) vs. those in all other roles combined rated items higher on every item, on average 0.4–0.6 points higher. These were significantly higher on 10 of 18 comparisons (including both PRISM context and RE-AIM outcomes) P<.01 and marginally significant on 5 others. Researchers (n = 133) gave similar or slightly lower ratings than those in other roles; only 1 of 18 comparisons was significant; the magnitude of differences were not large.	Clinicians saw intervention programs more positively (rated higher) than others Researchers’ ratings were similar to those in other roles
Research Question 3: How much congruence is there across team members?	Intraclass correlations (ICCs) for PRISM context variables: 0.14– 0.20, none were significant at p<.01 ICCs for RE-AIM outcomes tended to be somewhat higher: range of 0.09–0.44, Mdn = .23; 5 of 12 were significant at p<.01	Sample size restricted to 22 teams consisting of a total of 127 raters. Indicates low levels of agreement on how different team members rated the items; agreement somewhat lower for PRISM context items
Added Research Question:Mean scores on PRISM and RE-AIM indices acrossphases	On all 18 items, the average scoreon both PRISM context and RE-AIM outcome ratings were lower during implementation than during planning or sustainment phases. With one exception every comparison between the implementation phase and each of the other phases was significant at p<. 05 or less, usually at the p<.0001 level Scores for the planning and sustainment phases did not differ from each other	Largest and most consistent finding among all analyses conducted

## Data Availability

Data available from authors upon request.

## Abbreviations

TMF: Theories, Models, and Frameworks
IS: Implementation Science
PRISM: Practical Robust Implementation and Sustainability Framework
iPRISM: Iterative PRISM
RE-AIM Reach: Effectiveness, Adoption, Implementation, and Maintenance
ISI: Implementation and Sustainability Infrastructure
